# Keyword Index for Volume 96

**DOI:** 10.1038/sj.bjc.6603846

**Published:** 2007-06-12

13-*cis*-retinoic acid 424

14-3-3 296

^19^F-MRS 758

^1^H HRMAS NMR 1684

2-methoxyoestradiol 1368

4-phenylbutyrate 73

5-fluorouracil 21, 551, 701, 1043

5-FU and antifolates 769

abortion 1450

ABT-737 600

accident 752

acute leukaemia 535

adenocarcinoma 519

adenocarcinoma of the cardia 1767

adenocarcinoma of the oesophagus 1767

adenovirus 1871

adhesion 1699

adjuvant chemotherapy 1170, 1409, 1633

adjuvant treatment 1656

*ADRA1B* 383

advanced colorectal cancer 38

advanced non-small cell lung cancer 1644

aetiology 832

age–period–cohort pattern analysis 1767

*AIP* 352

Akt 445, 993, 1047

albumin 891

alcohol 821, 1469

allelic imbalance 499

amphiregulin 1569

amplification 474

anaplastic carcinoma 1549

androgen receptor 970, 1595

aneuploidy 1908

angiogenesis 1083, 1092, 1735, 1888

angiogenesis inhibitors 189, 1159, 1368, 1735, 1788

annexin V 928

anoikis 993

anthracyclines 226

anti-inflammatory agents 937

antimetabolites 61

antitumour activity 1204

antitumour immunity 617

antitumour response 1072

antivascular effect 1532

APC 631

APC mutations 1729

apoptosis 196, 231, 450, 575, 583, 639, 918, 928, 944, 1062, 1083, 1204, 1425, 1532, 1579, 1659

array analysis 762

array CGH 373

ASPP 196

atypical hyperplasia 1253

*β*-adrenergic receptor kinase (BARK1, GRK2) protein phosphatase (PP1*α*, PP2A) 82

B23 477

Barrett’s oesophagus 1377, 1767

basal cell carcinoma 523

BAT26 1409

BAX 1409

BBD 110

Bcl-2 1409, 1540, 1659

Bcl-2 inhibitor 600

bendamustine 1692

benign prostatic hyperplasia 137, 1475

benign prostatic hypertrophy 523

*BHD* 336

Big Blue 660

bile duct carcinoma 896

biliary tract carcinoma 896

biomarkers 118, 189, 362, 445, 857

birth order 1755

birth weight 134

bisphosphonates 255, 1526

bladder cancer 169, 1711

bladder tumours 762

Bmi-1 126

body mass index 49, 510, 845, 1457

body site 832

bone marrow 654, 1723

bone metastasis 1526

bone resorption 1716

borderline histology 1253

*BRAF* 445, 1549

brain 6

brain tumour 1293

BRCA1 11, 118, 1335

BRCA2 11, 1335

breakthrough pain 1828

breast cancer 11, 104, 134, 157, 162, 341, 352, 575, 639, 646, 654, 744, 801, 836, 841, 850, 903, 1001, 1092, 1135, 1197, 1204, 1258, 1368, 1404, 1436, 1462, 1504, 1520, 1526, 1625, 1743, 1747, 1796, 1802, 1808

breast feeding 815, 1450

breast neoplasms 1025, 1139, 1253

burden of disease 1484

*C. albicans* 137

Ca 110

CA125 1335

CA19-9 1650

cadherin 1

cancer cachexia 1216

cancer genetics 391

cancer immunotherapy 600

cancer networks 886

cancer plan 886

cancer registration 140, 1760

cancer registries 1493, 1743

cancer research agenda 875

cancer screening 623

cancer stem cell 201, 1020

capecitabine 912, 1348, 1514

capillaries 1788

carbonic anhydrase (CA IX) 104

carboplatin 559, 725

carcinoma 1425

cardia cancer 1272

cardiomyocytes 1667

cardioprotection 226

cardiotoxicity 937

cardiovascular disease 1747

care interval resolution 162

case–control studies 815, 1457

caspase-3 1659

caspase-7 944

caspases 450, 583

CBP 183

CCK-2R 1855

CD 8 lymphocytes 67

CD133 radial glia 6

CD326 417

CEACAM1 609

cell adhesion 1394

cell cycle 639

cell proliferation 477

cellular aging 1908

centrifugal assay for cell adhesion 1246

cerebral metastases 44

cervical cancer 143, 321, 591, 738, 1107, 1320, 1480

cervical intraepithelial neoplasia 738, 1234, 1419

cervical screening 1419

cetuximab 206

CGH 1237

Charlson comorbidity score 1462

chemiosensitivity 1358

chemoembolisation 49

chemoprevention 248

chemoradiation 1183, 1353

chemoradiotherapy 432, 912, 1650

chemoresistance 1699

chemosensitivity 241, 960

chemotherapy 44, 708, 732, 886, 1043, 1052, 1183, 1343, 1442, 1639, 1644, 1808

childhood cancer 815

childhood cancer survivors 1439

childhood leukaemia 1265

children 226, 667, 1147

China 172, 1554

cholangiocarcinoma 896

chromatin immunoprecipitation 1284

chromophobe renal cell carcinoma 336

chromosomal instability 960

chromosomal stability 1908

cigarette smoking 821

CIN2/3 1320

circulating nucleic acids 681

CIS 110

cisplatin 277

CK20 1711

clear cell carcinoma 290, 1613

clearance 1419

clinic 1625

clinical pharmacology 424, 725

clinical trials 189, 857, 1159

clodronate 1796

c-Met 329

CNS tumours 815

codon 72 1302

cohort 845, 1469

cohort studies 169, 510, 1457, 1755

colon cancer 61, 213, 701, 769

colon carcinoma 1409

colon carcinoma cells 21

colon carcinoma; uptake 1684

colonic neoplasm 1030

colonoscopy 828

colorectal 206, 692

colorectal cancer 140, 218, 231, 510, 660, 793, 821, 828, 986, 1112, 1118, 1166, 1329, 1750

colorectal liver metastases 222

colorectal metastases 1037

colorectal neoplasms 1605

colorectal tumours 352

combination studies 231

combined effects 255

combined modality 1183

combined modality therapy 1823

comparative genomic hybridisation 341, 667

complement system 67

consultation 875

controlled trial 744

core needle biopsy 1253

corpus cancer 1272

cost-effectiveness 206

CPT-11 38

C-reactive protein 222, 891

Cripto Mab 918

cryotherapy 738

CT chest 882

CT-26 1684

CTL 1072

cutaneous B-cell lymphoma (CBCL) 1540

*CXCR4* 485

cyclin-dependent kinase inhibitor 29

cyclooxygenase-2 575

CYP26 1675

cytoplasmic 970

cytosine deaminase 758

cytoskeleton 1

day 1+2 schedule 1692

DDR1 808

DDR2 808

death mechanisms 928

decatenation checkpoint 201

deletion 357

deliberate self-harm 752

deoxycytidine kinase 457

desmocollin 1783

desmoglein 1783

desmosome 1783

detection 1802

DHHC9 1896

diabetes 507, 1747

diagnosis 157, 535, 1197, 1329

diagnostic accuracy 1107

Dickkopf-1 (DKK1) 646

diet 248

differentiation 1204

disease susceptibility 523

distress 868

DNA double strand break 1707

DNA methylation 383, 1605

DNA mismatch repair 1605

DNA repair 118

DNA replication licensing 1384

DNA virus 137

DNA-strand breaks 231

docetaxel 432

dosing 424

doxorubicin 450, 937, 1520, 1667

drug evaluation 269

drug metabolism 424

dsRNA-dependent protein kinase 1216

dual targeting 952

ductal carcinoma 1404

dynamic contrast-enhanced magnetic resonance imaging 189

dysadherin 1404

dyskeratosis congenita 1020

dysplasia 492

early detection 738

early gastric cancer (EGC) 89

early-onset breast cancer 712, 1633

EBV 623

ED-B 1862

eEF1A2 1613

EGF-receptor signalling 408

EGFR 110, 284, 762, 793, 952, 1047, 1166, 1246, 1569

EGFR mutations 1191

EGR1 762

elapsed times 162

elderly patients 1197, 1823

EMT 1, 1783

endocrine tumour 49

endometrial cancer 134, 591, 1450, 1639, 1747

endoplasmic reticulum stress 944, 1062

endoscopic submucosal dissection (ESD) 89

endoscopy 492

England 1484

England and Wales 1135

environment 1740

EpCAM 417, 1013

ependymoma 6

EphB4 1083

epidemiological trends 1767

epidemiology 151, 157, 832, 1234, 1436, 1439, 1484

epidermal growth factor 1569

epidermal growth factor receptor (EGFR) inhibitor 1191

epigenetics 183

epirubicin 1043, 1633

epithelial ovarian carcinoma 314

epithelial–mesenchymal transition 1246

ER 583

erlotinib 857, 952

erythropoietin 692

ESFT 1914

Ewing’s sarcoma 1716

exposure patterns 523

expression 1237

extragonadal 667

faecal occult blood tests 218, 1750

familial breast cancer 1740

familial risk 1272

fascin 1118

E-cadherin 1404

fenretinide 1062

fertility and premature ovarian failure 1808

FGF 1544

FGFR4 1904

FHIT 110

fibrosis 1001

fluoropyrimidines 61

FOBT 218

follow up 1625

formaldehyde 1667

Foscan® 944

FOXp3 1879

fumarate hydratase 403

*γ*-inulin 67

G_2_ checkpoint 201

gallbladder cancer 1457

gallbladder carcinoma 896

gastric and intestinal marker 631

gastric cancer 95, 172, 277, 383, 1043, 1272, 1723

gastric carcinoma 631

gastric cardia 631

gastrin 464

gastroenteropancreatic tract 1178

gastrointestinal cancer 464

gastrointestinal stromal tumours (GIST) 776, 1656, 1834

gastro-oesophageal junction tumours 95

gefitinib 284, 762, 857, 1047, 1191

gemcitabine 73, 896

gemcitabine resistance 457

geminin 1384

gene amplification 1258

gene expression 1258

gene expression profiling 535

gene promoter methylation 1278

gene regulation 362

gene therapy 758, 1871

genetic assessment 391

genetic models of carcinogenesis 248

genetic testing 718

genomic instability 660

geographical incidence 1760

germ cell 667

germ cell tumour 357

germline 1265

gestational trophoblastic neoplasia 732

GIST 1834

glioblastoma multiforme 474, 1293

glutathione-*S*-transferase pi 1587

Gly388Arg polymorphism 1904

gonadal 667

gonorrhoea 169

Grb7 1520

gr/gr 357

GSK-3 1595

G-quadruplex 1223

*H. felis* 1855

HA14-1 600

HADS 868

HDAC inhibitor 73

head and neck cancer 1469

head and neck carcinoma 1569

health services 701

*Helicobacter pylori* 172, 1324

heparanase 1544

hepatitis B virus 1127

hepatitis C virus 1127

hepatocellular carcinoma 477, 1127

HER receptors 801

HER2 654

*HER2/neu* 485

hereditary leiomyomatosis and renal cell cancer 403

hereditary nonpolyposis 1605

heredity 1740

heterotypic cell–cell adhesion 1246

HHV8 183

HIF-1*α* 95, 1377

HIF-2*α* 1377

high LET radiation 1707

high-grade gliomas 1047, 1560

histology, twin births 1433

histone acetylation 1667

histone modifications 183

HMGA1 993

*HNF1β* 336

Hodgkin’s lymphoma 1442

hollow fibre assay 61

hormonal manipulation 1343

hormone 151, 845

HPA 609

HPV 514, 1554

HPV types 1480

HPV16/18 L1 VLPs 1320

HS 1544

HSULF-1 1544

human 1358

human immunodeficiency virus 1480

human kallikreins 6 362

human papillomavirus 143, 1234, 1419, 1480

hypoxia 104, 1302, 1377, 1888

hypoxia-inducible factor 403, 1284

hypoxia-inducible factor-1 (HIF-1) 1871

hypoxia-response element (HRE) 1871

ICRF-193 201

IDO 1879

*IGF1* genotype 712

*IL-6* gene 474

image analysis 189, 329

imatinib 1656

imatinib mesylate 1834

immune surveillance 1849

immunocytochemistry 329, 1107

immunohistochemistry 306, 314, 485, 1118, 1896

immunorecognition 1072

immunotherapy 1013, 1293

*in vivo* pharmacodynamics 61

incidence 818, 1772

incidence rates 1743

indisulam 559

individual patient data meta-analysis 1170

individualized chemotherapy 241

individualized therapy 857

infection 169

inflammation 169, 937

inflammatory response 222

inhibition 1216

InsGas 1855

insulin-like growth factor binding protein 3 1587

integration 1554

integrin 1394

interfering RNA 464

international comparisons 1493

intestinal metaplasia 383

intravenous morphine 1828

invasion 1, 1112

IP-10 1735

irinotecan 21, 38, 439, 546, 551, 912, 1514

isotretinoin 424

keratinocytes 126

Ki-67 445, 970, 1504, 1711

kidney cancer 403, 646

kidney neoplasms 845

*KIT* 1656

*KRAS* 631, 1166

KSHV 183

L1 609

L19-SIP 1862

LASP-1 296

leucovorin 1043

lipopolysaccaride 262

liver function tests 1178

liver metastases 1112

liver resection 1037

lobular carcinoma 1404

locally advanced rectal cancer 551, 912

LOH 499

lomustine 44

long-term 1135

long-term outcome 1324

low dose 1707

low dose rate 1707

Lugol staining 492

lung cancer 519, 646, 808, 886, 1052, 1278, 1904

lymph node 321

lymph node resection 1817

lymphangiogenesis 541, 575, 1092

macrophage 1716

magnesium 510

malignancies 477, 1101

malignant melanoma 832

mammography 157

MAP kinase 445

mass screening 56

mathematical model 143

Matrigel plug assay 1368

mayo regime 701

MBD4 660

Mcm2 1384, 1711

MDR 918

meat 1139

medication review 744

meeting report 417

MEK5/ERK5 1384

melanoma 44, 445, 1072, 1772, 1879

melanoma model 609

menopause 841

meta-analysis 1127, 1457, 1504, 1796

metachronous recurrent cancer 89

metalloproteinase 783

metaplastic 1855

metastases 1723

metastasis 262, 541, 903, 1394

metastatic colorectal cancer 439, 546

methylation 183, 1587

methylcholanthrene 1849

MGMT 960

microarray analysis 535

microarrays 341, 1155, 1896

microcirculation 692

micrometastases 321

micrometastasis 654

microsatellite instability 89, 631, 1409, 1605, 1896

microtubule 1532

microvessel density 1112

migration 296, 1560

minichromosome maintenance proteins 1107

minisatellite 1265

mitochondrial membrane potential 928

mitomycin 1052

mitotic arrest 1532

MLPA 1914

MMP 903

modelling 514

molecular markers 1166

molecular profiling 321

monoclonal antibody 408

monoHER 450, 937

mortality 1747, 1772

Motzer 567

mouse tumours 67

mTOR 952

mucosa-associated lymphoid tissue lymphoma 1324

multidrug resistance 1579

multiparity 712

multiple colorectal adenoma 1729

multiple primaries 529

muscle protein degradation 1216

muscle protein synthesis 1216

mutation 336, 808, 1166, 1265, 1549, 1656

mutation frequency 660

MYC 82

MYH/MUTYH mutations 1729

N^*ε*^-(carboxymethyl)lysine 937

nasopharyngeal carcinoma 617

NCAM 1699

necrosis 928

neoadjuvant chemotherapy 341, 1037

neoplasm staging 1030

neoplasms 667

neovascularization pathologic 980

neuroblastoma 1675, 1699

neuro-ectodermal tumours 1062

neuroendocrine carcinomas 1178

neuroendocrine tumours 1343

neutralising antibody 1320

NK cells 1839

NKT cells 600

(non) tumour cells 450

non-phosphorylated peptide 1520

nonseminoma 529

non-small cell lung cancer 886, 1052, 1191, 1569

NPC 623

nucleophosmin 477

nucleoside transporter 457

number of sibling 1755

nurse’s role 1057

obesity 1457

oesophageal cancer 172, 432, 708, 1348, 1823

oesophageal squamous cell carcinoma 1554

oesophagitis 492

oestrogen receptor 1595

offspring’s gender 1436

oncogenic virus 183

opioids 1828

oral cancer 126, 1425

oral carcinoma 818

oro-pharyngeal carcinoma 818

osteoclast 1716

osteosarcoma 255

OTFC 1828

ovarian cancer 11, 151, 290, 296, 362, 485, 850, 1083, 1433, 1544, 1747, 1817

ovarian carcinoma 306, 1335

ovarian neoplasms 1908

ovarian reserve 1808

ovarian tumour 1613

oxaliplatin 38, 231, 439, 546, 1043, 1348

oxygenation 692

p14^ARF^ 1914

p16 1914

p16^INK4A^ 126, 306

p21^Cip1^ 970

p300 183

p53 196, 277, 631, 769, 1302, 1425

*p53* mutation 492

p53/BAX pathway 1409

p53-response element 1579

paclitaxel 241

paediatric cancer 1493

paediatrics 725

PALGA 1234

pancreatic adenocarcinoma 993

pancreatic cancer 373, 457, 507, 1183, 1353, 1358, 1650

pancreatic carcinoma 73

PAP 352

papillary carcinoma 1549

papillary thyroid cancer 16

parity 845

participatory 875

paternal/maternal lineage 1740

PCNA 110, 477

*PDGFRA* 1656

PDT 1839

penetrance 11

peripheral blood 1723

P-glycoprotein 918

pharmacokinetics 559, 1692

pharmacy 744

phase I 21, 29, 559, 1353, 1692

phase II study 432, 864

phospholipids 1684

photodynamic therapy 67, 944

Physiology Score 213

PI3K pathway 1595

Pidd 1425

pituitary adenoma 352

PL 583

plakoglobin 1783

plasma/serum DNA 681

plasma/serum RNA 681

platinum compounds 896

platinum–DNA adducts 725

podoplanin 1

polycomb 126

polymorphism 336, 1302

population-based 1462, 1475, 1493, 1743

POSSUM 213

postmenopausal 151

post-treatment parenthood 1442

potentiation 269

pRb 306

precursor 492

predictive 1155

predictive marker 457

predictive value 1253

pre-eclampsia 1436

pre-malignant 1855

preoperative chemoradiation 551

preoperative chemoradiotherapy 1498

prevalence 868

prevention 828

primary brain lymphoma 864

primary breast cancer 891

primary health care 1057

primary prevention 1057

primary tumour cells 241

priorities 875

prodrugs 1667

profile 1237

progestagen 841

progesterone receptor 241

prognosis 306, 314, 474, 639, 801, 903, 986, 1030, 1101, 1118, 1384, 1409, 1904, 1914

prognostic 1155

prognostic and diagnostic marker 646

prognostic marker 104

prognostic value 1504

projections 1484

proliferation 970

proportional incidence method 1750

prospective studies 1139

prostate adenocarcinoma 269

prostate cancer 82, 137, 499, 523, 1384, 1475, 1587, 1595

prostate neoplasms 56

prostate tumours 352

prostate-specific antigen 56, 970

prostatic hyperplasia 980

prostatic intraepithelial neoplasia 1587

prostatic neoplasms 980

protection 1320

protein expression 269

proteomics 284

psychological; BRCA1/2 718

Pt-DNA adducts 231

PTHrP 1394

PTK6 (BRK) expression 801

quality assurance 157

quality of life 1834

R116010 1675

radiation 1439

radiation injury 1001

radioimmunotherapy 1862

radiosensitisation 1532

radiosensitivity 118

radiosensitizer 1650

radiotherapy 692, 708, 1183, 1442, 1871

RAMBAs 1204

randomised clinical trials 1170

randomised controlled trials 56, 1025

RAS 16

real-time polymerase chain reaction 783

rectal cancer 213, 1170

recurrence 1625, 1802

Recurrent glioblastoma 960

recurrent tumours 321

regulatory T cells 1849

relapse 732, 1147

relative risk 752

relative survival 1135

renal carcinoma 1284

renal cell carcinoma 567, 1888

reproduction 1450

reproductive factors 845

resveratrol 1595

retinoic acid 1204, 1675

RET/PTC oncogene 16

retrospective review 1802

RHPS4 1223

ribonucleotide reductase 457

rice bran 248

risk 1450

risk factors 1139, 1147

risk management 718

rituximab 1540

RKIP 1540

RNA interference 126

ROC curve analysis 793

role in cancer 417

S-1 277, 1353, 1650

salivary glands 1101

*Salmonella typhimurium* 758

salvage therapy 864

SCCHN 408

scid mouse 609

SCLC 886

scoring system 793

Scotland 818, 832, 1772

screening 140, 143, 218, 738, 868, 1107, 1335, 1750

*SDF-1* 485

*SEC11L3* 373

second-line therapy 1052

‘see and treat’ 738

seminoma 529

senescence 1707

sensitivity 1750

sequential 1644

serine protease 362

serology 623

serum 1278

service users 875

sex of children 1433

sex ratio 1439

sibship size 1755

sigmoidoscopy 828

signal transduction 284

signalling transduction pathways 918

Singapore Chinese 821

single nucleotide polymorphism 783

SKOV-3 296

small cell 708

smoking 845, 1191

smoking cessation 1057

SN-38 21

SNP 499

socioeconomic background 836

socioeconomic deprivation 1760

socio-economic status 818

sonic hedgehog 1855

SOX2 1293

specificity 56

spheroids 1072

sputum 1278

squamous cell carcinoma 519, 952, 1234

stage at diagnosis 836

stage III NSCLC 1498

staging investigations 882

standardised incidence ratio 1475

Stat3 591

stem cells 6, 1020, 1223

stomach neoplasms 1514

stool test 1329

streptozotocin 49

subcellular localisation 944

suicide 752

sulphamates 1368

sunlight exposure 832

superior sulcus tumour 1498

surgery 262, 1025

surrogate marker 284

survival 213, 222, 519, 529, 836, 891, 1083, 1147, 1462, 1772, 1796, 1802, 1817, 1834

survival analysis 1030

survivin 639

survivin-*Δ*Ex3 1659

Sweden 519

systematic review 226, 391

systemic inflammatory response 891

T cells 1839

tamoxifen 850, 1025

targeted intervention 162

targeted therapy 1520

TAS-102 61

Taxol 1223

technician 744

teenage and young adult cancer 1760

tegafur–uracil 38

telangiectasia 1001

telomerase 1020, 1223

telomere 1020, 1223, 1908

temozolomide 44, 864, 960

temsirolimus 952

testicular cancer 357, 529

testicular seminoma 882

tetracyclines 1526

TGF-*β* 1560

TGF*β*1 1001

TGF*β*2 immunosuppression 1879

therapeutic monitoring 725

thymidylate synthase 277, 769

thyroid 1237, 1549

TIMP 903

tissue arrays 903

tissue factor 290

tissue microarray 82, 329, 485, 591, 776, 793

tissue microdissection 373

tolerogenic dendritic cell 1879

tomography 1030

topoisomerase II 201

toxicity 701

*TP53* 336

transcription 1284, 1394

transforming growth factor- alpha 1569

translation elongation 1613

trastuzumab 654, 1520

treatment 836, 1197, 1834

Treg 617

trends 1135, 1743

trial 1796

trifluorothymidine 231

trifunctional antibodies 1013

trimodality 1498

tubulin 1684

tumour 6, 1278, 1862

tumour characteristics 1197

tumour escape 980

tumour hypoxia 1871

tumour immunity 1839

tumour infiltrating lymphocytes 617

tumour location 1324

tumour M2-PK 1329

tumour marker 623, 681

tumour vascularisation 49

tumoural invasion 903

tumour-associated antigen 567, 1293

tumour-targeted superantigen 567

TUNEL 1101

TVU 1335

Twist 314

type 1419

tyrosine kinase inhibitor 408

TZT-1027 1532

UFT 38, 439, 1170

ultraviolet radiation 523

urinary 5-hydroxyindoleacetic acid 1178

urokinase plasminogen activator (u-PA) 262

uterine cervical cancer 1735

uterine cervical neoplasms 1057

vaccination 143, 514

vaccines 1320

validation 1155

valproic acid 1699

vascular disrupting drugs 1159

vascular endothelial cell growth factors 1092

vascular endothelial growth factor receptor-1 1723

vasculature 1862

VEGF 776, 1377, 1735

VEGF signalling 1788

VEGF-A 1092

VEGF-C 541, 1092

VEGF-D 1092

VEGFR-3 541

venous thromboembolism 290

Versican 1560

vimentin 986

vinorelbine 1052, 1633

viral load 1554

von Hippel–Lindau 1284

waiting times 162

WHO classification 1178

women 1135

WTH3 gene 1579

WXC-340 262

xenograft human tumour 758

X-ray computed 1030

XRCC1 1001

Y chromosome 357

young patients 1743

young-onset diabetes 507

Zambia 1480

zoledronic acid 255

zyxin 296

